# Elucidation of a Novel Role of YebC in Surface Polysaccharides Regulation of *Escherichia coli bipA*-Deletion

**DOI:** 10.3389/fmicb.2020.597515

**Published:** 2020-11-09

**Authors:** Eunsil Choi, Hyerin Jeon, Changmin Oh, Jihwan Hwang

**Affiliations:** ^1^Microbiological Resource Research Institute, Pusan National University, Busan, South Korea; ^2^Department of Microbiology, Pusan National University, Busan, South Korea

**Keywords:** GTPase, YebC, cold shock, capsule, BipA, lipopolysaccharide (LPS)

## Abstract

The BipA (BPI-inducible protein A) protein is ubiquitously conserved in various bacterial species and belongs to the translational GTPase family. Interestingly, the function of *Escherichia coli* BipA is not essential for cell growth under normal growth conditions. However, cultivation of *bipA*-deleted cells at 20°C leads to cold-sensitive growth defect and several phenotypic changes in ribosome assembly, capsule production, and motility, suggesting its global regulatory roles. Previously, our genomic library screening revealed that the overexpressed ribosomal protein (r-protein) L20 partially suppressed cold-sensitive growth defect by resolving the ribosomal abnormality in *bipA*-deleted cells at low temperature. Here, we explored another genomic library clone containing *yebC*, which encodes a predicted transcriptional factor that is not directly associated with ribosome biogenesis. Interestingly, overexpression of *yebC* in *bipA*-deleted cells diminished capsule synthesis and partially restored lipopolysaccharide (LPS) core maturation at a low temperature without resolving defects in ribosome assembly or motility, indicating that YebC may be specifically involved in the regulation of exopolysaccharide and LPS core synthesis. In this study, we collectively investigated the impacts of *bipA*-deletion on *E. coli* capsule, LPS, biofilm formation, and motility and revealed novel roles of YebC in extracellular polysaccharide production and LPS core synthesis at low temperature using this mutant strain. Furthermore, our findings suggest that ribosomal defects as well as increased capsule synthesis, and changes in LPS composition may contribute independently to the cold-sensitivity of *bipA*-deleted cells, implying multiple regulatory roles of BipA.

## Introduction

BipA (also known as TypA) is a GTPase belonging to translation/elongation factors (TRAFAC) family and shares structural similarity with translational GTPases (trGTPases), such as IF2, EF-Tu, EF-G, EF4, and RF3 (Ero et al., 2016). It is widely distributed in bacteria and plants (Margus et al., 2007; Wang et al., 2008). Like other ribosome-associated trGTPases, BipA binds to the ribosome only in the GTP-bound form, which in turn stimulates its GTP hydrolysis activity (deLivron and Robinson, 2008; deLivron et al., 2009; Kumar et al., 2015; Choi and Hwang, 2018).

While most trGTPases function as translation factors, BipA has been implicated as a ribosome assembly factor. In *Escherichia coli*, the deletion of *bipA* causes defects in growth and 50S ribosomal subunit assembly at low temperature (Choi and Hwang, 2018). *E. coli bipA* mutant exhibits the accumulation of 44S particles, precursors of 50S ribosomal subunits lacking L6 ribosomal protein (r-protein) and containing immature 16S and 23S rRNAs. Furthermore, the cold-sensitivity exhibited by *bipA*-deleted cells is suppressed by deletion of *rluC*, which encodes a pseudouridine synthase that introduces pseudouridine into U955, U2504, and U2580 of 23S rRNA located near the binding site of BipA (Conrad et al., 1998; Krishnan and Flower, 2008; Kumar et al., 2015). In addition, it was recently revealed that overexpressed r-protein L20 partially restores growth and ribosome maturation in a *bipA*-deleted strain at low temperature (Choi et al., 2019). These genetic links between BipA and RluC or L20 clearly support the notion that BipA plays a pivotal role in 50S ribosomal subunit maturation.

Interestingly, the function of BipA has also been implicated in various stress responses as its expression is significantly increased in response to stresses, such as low temperature, oxidative stress, antibiotics, and antimicrobial peptides (Qi et al., 1995; Farris et al., 1998; Barker et al., 2000; Pfennig and Flower, 2001; Wang et al., 2008; Neidig et al., 2013). In contrast, *bipA* is not essential for survival under normal growth condition (Pfennig and Flower, 2001). In *Pseudomonas aeruginosa*, deletion of *bipA* results in increased sensitivity to antibiotics such as meropenem, ceftazidime, tetracycline, polymyxin B, and colistin (Neidig et al., 2013). In *Salmonella typhimurium*, expression of *bipA* is induced by BPI (Bactericidal/permeability-increasing protein), which is a cationic antimicrobial protein produced by human neutrophils (Weiss and Olsson, 1987; Qi et al., 1995). Recently, we have shown that in non-pathogenic *E. coli* K-12 strain, cAMP-receptor protein (CRP) promotes the expression of *bipA* at low temperature (Choi and Hwang, 2018).

Unlike BipA in non-pathogenic *E. coli*, BipA in enteropathogenic *E. coli* (EPEC) is post-translationally regulated by phosphorylation of tyrosine residues enhancing GTPase activity. This BipA promotes the expression of two pathogenicity islands, *espC* and locus of enterocyte effacement (LEE), through positive regulation of LEE-encoded regulator (Ler) transcription (Farris et al., 1998; Freestone et al., 1998; Grant et al., 2003). In addition, a *bipA*-deleted EPEC mutant exhibited hypermotility phenotypes at 37°C (Grant et al., 2003). As a consequence, *bipA*-deleted EPEC strains lose the ability to induce rearrangement of the actin cytoskeleton, which is a target of bacterial virulence factors (Farris et al., 1998; Sal-Man et al., 2009). The EPEC *bipA* mutant exhibits increased transcription of group 2 capsule gene clusters at 20°C, suggesting that BipA represses K5 capsule production at 20°C (Rowe et al., 2000). Paradoxically, at 37°C, the level of these transcripts decreases in this *bipA* mutant, indicating that BipA is required for their maximum transcription at 37°C (Rowe et al., 2000). More peculiarly, however, the *bipA*-deleted K-12 strain presents decreased motility and overproduction of colanic acid (CA)-containing capsule at low temperature (Choi and Hwang, 2018), which is contrary to phenotypes in EPEC, implying that BipA may function differentially in EPEC and K-12 strains. Regarding lipopolysaccharide (LPS), an insertional mutation at the 3′ end of *bipA* was screened as a suppressor mutant for the *E. coli* K-12 *waaQ* mutation, which inactivates the entire *waa* operon and confers hypersensitivity to sodium dodecyl sulfate (SDS), bile salts, and the hydrophobic antibiotic novobiocin through impairment of LPS inner core biosynthesis (Moller et al., 2003). This insertional *bipA* mutant was partially functional and somehow restored LPS core biosynthesis by expressing the *waa* operon downstream of *waaQ* (Moller et al., 2003). In *P. aeruginosa* PAO1, transposon insertion in *bipA* leads to reduction in swarming motility and impairment of biofilm development (Overhage et al., 2007). In addition, deletion of *bipA* in *P. aeruginosa* PA14 causes a reduction in virulence in a *Dictyostelium discoideum* amoeba model (Rahme et al., 1995; Choi et al., 2002; Neidig et al., 2013). This PA14 mutant also exhibits reduction in the expression of Type III secretion system (T3SS) genes and impairment of biofilm formation (Neidig et al., 2013). Thus, it is likely that BipA is a crucial virulence factor regulating capsule, LPS, motility and biofilm formation.

In our previous study, we conducted genomic library screening in an *E. coli* K-12 *bipA* mutant to further comprehend its physiological role. The *yebC* gene was screened as a suppressor of cold-sensitivity (Choi et al., 2019). The YebC/PmpR family proteins are widespread and conserved in many bacteria (Wei et al., 2018). Even though its function could not be deduced from the X-ray structure of *Aquifex aeolicus* YebC (Shin et al., 2002), recent evidence supports the notion that in various bacteria, YebC is a potential transcriptional regulator with either activator or repressor function. For example, in *Alishewanella* sp. WH16-1, YebC induced by Cr(VI) positively regulates the transcription of *ruvRCAB*, contributing to Cr(VI), As(III), Sb(III), and Cd(II) resistance (Wu et al., 2019). In *Lactobacillus delbrueckii* subsp. *lactis* CRL 581, transcription of *prtL*, *oppA*, and *optS* is repressed by binding of YebC to the respective promoter regions (Brown et al., 2017). Interestingly, YebC of *Edwardsiella piscicida* binds to *edwR* or *ETEA_0873* promoter regions resulting in the repression of quorum-sensing (QS) response regulator or activation of T3SS expression, respectively (Wei et al., 2018). In *P. aeruginosa*, YebC inhibits the transcription of *pqsR*, which is a *Pseudomonas* quinolone signal QS response regulator (Liang et al., 2008). These accumulated data raise the possibility that YebC may control the transcription of specific genes.

Despite the possible roles of YebC as a transcription factor in various bacteria, the function of YebC in *E. coli* has not been evaluated yet. Therefore, in the present study, considering pleiotropic phenotypes such as capsule production, LPS synthesis, biofilm formation, and motility altered by *bipA*-deletion, we explored the phonotypic impacts of overexpression of *yebC* on a *bipA*-deletion genetic background to elucidate the role of YebC.

## Materials and Methods

### Bacterial Strains and Growth Conditions

The *E. coli* strains used in this study are listed in Table 1. The MG1655 *yebC*::*kan* (ESC48) and *rcsF*::*kan* (ESC53) were constructed by bacteriophage P1 transduction using JW1853 (*yebC*::*kan*) and JW0192 (*rcsF*::*kan*), respectively (Baba et al., 2006). The P1 lysate obtained from JW0192 was also employed to construct a double mutant (ESC54; Δ*bipA*, *rcsF*::*kan*). Kanamycin resistant cells were isolated and the disruption of each gene was confirmed by PCR using the primer sets listed in Supplementary Table S1. The removal of the kanamycin cassette at the *bipA* locus was carried out as described previously (Datsenko and Wanner, 2000). All *E. coli* strains were cultivated at either 37°C or 20°C in Luria–Bertani (LB) broth with chloramphenicol (50 μg/ml), kanamycin (50 μg/ml), or ampicillin (100 μg/ml) as needed. Bile salts was purchased from Sigma-Aldrich (catalog number: 48305).

**TABLE 1:** Strains and plasmids used in this study.

Strains	Description	Source or references
MG1655	F^–^ λ^–^ *ilvG*^–^ *rfb*-*50 rph*-*1*, *Escherichia coli* K-12	Blattner et al., 1997
DH5α	F^–^ λ^–^ Φ80*lac*ZΔM15 Δ(*lac*ZYA-*arg*F) U169 *recA*1 *endA*1 *hsdR*17(rk^–^, mk^+^) *phoA supE*44 *thi*-1 *gyrA*96 *relA*1	Gibco-BRL
ESC19	*bipA*::kan, MG1655	Choi and Hwang, 2018
ESC48	*yebC*::kan, MG1655	This study
ESC53	*rcsF*::kan, MG1655	This study
ESC54	*rcsF*::kan, Δ*bipA*, MG1655	This study
JW1853	*yebC*::kan, BW25113	Keio collection
JW0192	*rcsF*::kan, BW25113	Keio collection
**Plasmids**		
pACYC184	Cm^R^, Tc^R^, *ori* p15A, cloning vector	New England Biolabs
pACYC184BipA	*bipA*^+^, pACYC184	Choi and Hwang, 2018
pACYC184BipA_N128D_	*bipA* (N128D), pACYC184	Choi and Hwang, 2018
pBIS02-2	*rpmI^+^-rplT^+^-pheM^+^*, pACYC184	Choi et al., 2019
pBIS05	*nudB^+^-yebC^+^-ruvC^+^*, pACYC184	This study
pBIS05-1	*nudB*^+^, pACYC184	This study
pBIS05-2	*nudB^+^-yebC^+^*, pACYC184	This study
pBIS05-3	*nudB^+^-yebC^+^-ruvC^+^*, pACYC184	This study
pBIS05-stop	*nudB^+^-yebC-UAG_1_-ruvC^+^*, pACYC184	This study

### Construction of an *E. coli* Genomic Library and Suppressor Screening

Genomic library construction and suppressor screening were performed as described in our previous study (Choi et al., 2019). In brief, genomic DNA from *bipA*-deleted cells (ESC19) was purified and partially digested DNA fragments were ligated into pACYC184 yielding the ESC19 genomic library. After transforming ESC19 cells with the genomic library, suppressors of cold-sensitive growth were screened by selecting larger colonies than those harboring empty vector (pACYC184). To confirm their suppressing activities, library clones extracted from those larger colonies were retransformed into ESC19 cells. A total of 26 isolated library clones were sequenced from both ends to identify the inserted genomic DNA fragments.

### Plasmid Construction

To construct truncated library clones of pBIS05, DNA fragments of pBIS05 were amplified by PCR using pBIS05 as a template and the primer sets presented in Supplementary Table S1. The PCR fragments were ligated into the *Sma*I site of pUC19 followed by digestion with *Bam*HI. Each digested DNA insert was ligated into the *Bam*HI site of pACYC184, yielding pBIS05-1, pBIS05-2, and pBIS05-3. To replace the initiation codon AUG of *yebC* with stop codon UAG, site-directed mutagenesis PCR was carried out using pBIS05 as the template and the primer set in Supplementary Table S1.

### Sucrose Density Gradient Sedimentation

Polysome and subunit profiling experiments were carried out as described previously (Choi and Hwang, 2018). In brief, MG1655, ESC48, or ESC19 transformants harboring plasmid were cultured to the logarithmic phase at 37°C or 20°C in LB medium supplemented with the appropriate antibiotics. To trap polysomes, chloramphenicol was added to cultures at a final concentration of 100 μg/ml (for the strains MG1655 and ESC48) or 150 μg/ml (for the ESC19 transformants). After an additional 3 min of incubation, the cells were harvested by centrifugation. The cell pellets were resuspended in Buffer BP [20 mM Tris–HCl (pH 7.5), 10 mM MgCl_2_, 100 mM NH_4_Cl, and 5 mM β-mercaptoethanol (BME)]. Cells were then lysed by freeze–thaw cycle, and the cell lysates were obtained by centrifugation. Then, cleared lysates were loaded into 5–40% sucrose gradients in Buffer BP and resolved by ultracentrifugation at 4°C using a Beckman SW41 rotor at 170,000 × *g* for 2.5 h. For ribosomal subunit profiling experiments, the cells were lysed in Buffer BS [20 mM Tris–HCl (pH 7.5), 1 mM MgCl_2_, 100 mM NH_4_Cl, and 5 mM BME], and cleared cell lysates were prepared similarly as described for polysome profiling experiments. Then, lysates were subjected to 5–25% sucrose gradients in Buffer BS followed by centrifugation at 4°C in the same rotor for 3.5 h at 170,000 × *g*.

### Observation of Cell Morphology and Capsule Staining

The morphology of colonies grown for 18 h at 37°C or for 96 h at 20°C on LB agar plates was observed using Azure C200 photo documentation (Azure, United States). For capsule staining, 5 μl of the resuspended colonies in 0.4% saline solution were dropped on a clean glass microscope slide and mixed with 10 μl of 1% crystal violet solution. Then, the mixture was spread using the edge of a clean cover glass to form a thin smear by placing the end of cover glass at an angle to the end of the slide containing the cells. After air drying for 10 min, crystal violet was washed with 20% copper sulfate solution. Then, to stain the background around *E. coli* cells, 1% Congo red solution was added to the extent that the smear was sufficiently covered. After staining for 10 min, Congo red was gently rinsed away with distilled water followed by an additional air drying. Then, capsule was observed under oil immersion with an Axio Observer (ZEISS, Germany) light microscope at 1,000 × magnification.

### qRT-PCR

Colonies that formed on LB agar plates were scraped using 10 ml of fresh LB medium, and the collected cells were centrifuged to remove supernatant. Total RNA extraction and cDNA synthesis were carried out as we previously described (Choi and Hwang, 2018). We performed qRT-PCR using 1 μl of cDNA and 2 pmole of each gene-specific primer (Supplementary Table S1) in a 20 μl volume with 2× SYBR Green master mix (Qiagen). The reactions were carried out on a QuantStudio 3 Real-Time PCR system (Applied Biosystems) using the following cycling parameters: 95°C for 15 min and 40 cycles of denaturation at 95°C for 15 s, primer annealing at 55°C for 30 s, and extension at 72°C for 30 s. The *gapA* gene served as an endogenous reference (Navasa et al., 2011), and relative quantification (RQ) values were calculated using the comparative C_t_ method (Livak and Schmittgen, 2001).

### Isolation of LPS and Silver Staining

Lipopolysaccharide was extracted from *E. coli* strains using the hot-phenol and diethyl ether method as previously described (Davis and Goldberg, 2012). Cells grown overnight in 3 ml of LB containing appropriate antibiotics were spread on LB agar plates. After incubation at 37°C or 20°C, colonies were scraped using 10 ml of fresh LB medium. The pelleted cells were resuspended in 200 μl of 1× SDS buffer [50 mM Tris–HCl (pH 6.8), 2% BME, 2% sodium dodecyl sulfate (SDS), and 10% glycerol] and boiled in a water bath for 15 min. Samples were cooled at room temperature for 15 min. Five microliters each of DNase I (10 mg/ml) and RNase A (10 mg/ml) solutions were added to the sample, and these mixtures were incubated at 37°C for 30 min. Next, 10 μl of Proteinase K (10 mg/ml) was added, followed by another incubation at 59°C for 3 h. 200 μl of ice-cold Tris–saturated phenol was added to each sample, and the samples were briefly vortexed for 5 to 10 s. The phenol mixtures were incubated at 65°C for 15 min, vortexing occasionally. After cooling at room temperature, 1 ml of diethyl ether was added to each sample, and the mixture was vortexed for 5 to 10 s. The samples were centrifuged at 17,000 × *g* for 10 min, and LPS was carefully extracted from the bottom layer. LPS samples were separated using 12.5% Tricine SDS-PAGE and visualized by silver staining as previously described (Morrissey, 1981).

### Biofilm Staining

Quantification of biofilm formation in glass tubes was performed as described previously (Naves et al., 2008). Twenty-five microliters of overnight culture were inoculated into 2.5 ml of fresh LB in glass tubes (16 × 100 mm). The cells were incubated for 24 h at 37°C or 48 h at 20°C, followed by removal of the culture medium. The tubes were rinsed once with 2.7 ml of 0.85% saline solution. After drying at 37°C for 30 min, 2.7 ml of 0.1% crystal violet dye was added to each glass tube for 5 min at room temperature. After this, the tubes were washed three times with 3 ml of distilled water, and the excess water was removed from the glass tubes by inverting the tubes and tapping them on paper towels. The glass tubes were allowed to dry and then photographed. The crystal violet was solubilized by adding 3 ml of 95% ethanol to the glass tube. Absorbance at 540 nm was then measured and recorded using a Multiskan^TM^ GO Microplate Spectrophotometer (Thermo Fisher Scientific^TM^). Tubes containing only LB were used as controls, and their OD_540_ values were subtracted from all samples.

### Statistical Analysis

Results are presented as the mean ± SD of three independent experiments. The data were analyzed by unpaired two-tailed *t*-test. The statistical analyses were performed with the following significance levels: NS, non-significant; ^∗^*p* < 0.05; ^∗∗^*p* < 0.01; ^∗∗∗^*p* < 0.001.

## Results

### Screening for Multi-Copy Suppressors of Cold-Sensitivity in a bipA-Deleted Strain

**FIGURE 1:**
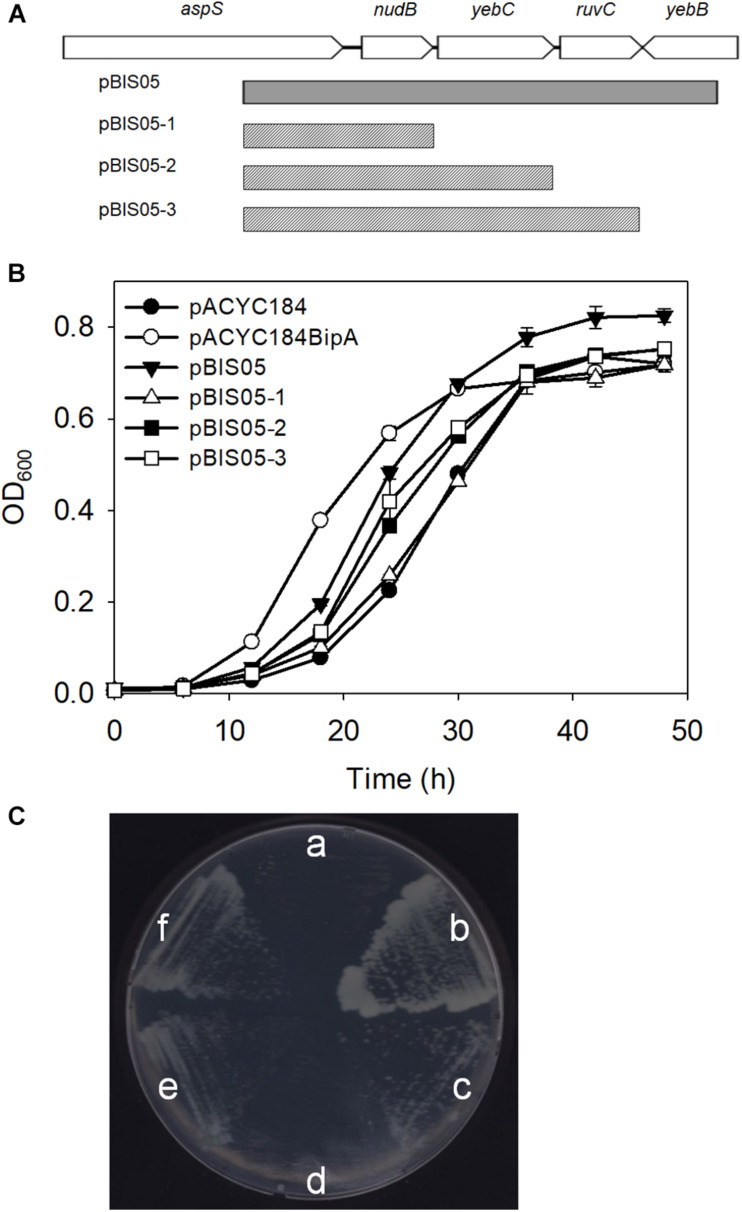
Genomic library clone that suppresses cold-sensitive growth defects of the ESC19 strain at 20°C. **(A)** Schematic diagram of the genomic locus containing the suppressor gene. The gray square box represents the suppressor library clone. The scratched boxes represent the truncated clones constructed from pBIS05. (**B**,**C**) Recovered growth phenotype of the ESC19 strain caused by the isolated suppressor. ESC19 cells were transformed with each plasmid as presented above. Each transformant was incubated in LB medium containing chloramphenicol and kanamycin at 20°C. Cultures were diluted five times before measurement of the optical density at 600 nm. Experiments were performed in three independent replicates and error bars represent SD. Overnight cultures were diluted 100-fold with LB medium containing chloramphenicol and kanamycin. LB agar plates supplemented with same antibiotics were streaked with 2.5 μl of diluted culture. **(a)**, pACYC184; **(b)**, pACYC184BipA; **(c)**, pBIS05; **(d)**, pBIS05-1; **(e)**, pBIS05-2; and **(f)**, pBIS05-3.

Previously, we constructed a genomic library using genomic DNA from the strain ESC19 (*bipA*-deletion) and searched for an element(s) in the *E. coli* genome that can revert cold-sensitivity in the strain ESC19. A total of 26 library clones were isolated as positive suppressor clones. We have shown that among them, overexpression of *rplT*, whose gene product is r-protein L20, partially rescued the ribosomal defects caused by the deletion of *bipA* at low temperature. Among the remaining clones that were screened, we attempted to investigate the suppressor clone, pBIS05, which contains the *nudB*-*yebC*-*ruvC* genomic DNA fragment. Using this clone, three truncated clones, pBIS05-1, pBIS05-2, and pBIS05-3, were constructed to identify the gene that was responsible for suppression (Figure 1A). Then, these clones were transformed into the ESC19 cells, and the transformants were tested for their abilities to grow normally at a non-permissive temperature (20°C) on LB medium containing chloramphenicol and kanamycin. As shown in Figure 1B, cells harboring pACYC184 had a longer lag phase compared to ESC19 cells with pACYC184BipA. Nevertheless, the growth rates of these two transformants in the mid-exponential phase appeared to be similar. The lag phase of ESC19 cells harboring pBIS05, pBIS05-2, or pBIS05-3 was shorter than that of ESC19 cells with pACYC184, while their growth rates were in a similar range to those of the two control strains. Notably, the ESC19 cells transformed with pBIS05-1 grew as poorly as the cells harboring pACYC184 (Figure 1B). The replacement of *yebC* start codon in pBIS05 with a stop codon led to a loss of its suppressive activity, indicating that YebC is a responsible suppressor protein (Supplementary Figure S1). These suppressive phenotypes were further confirmed in solid medium at 20°C. In Figure 1C, the pattern of colony formation ability of ESC19 cells transformed with pBIS05 variants indicated very similar suppressive effects as in Figure 1B, indicating that the *yebC* gene is indeed a suppressor gene. Despite this suppression activity, it should be noted that the suppression was partial.

### Inability of the Suppressor to Resolve Ribosomal Defects in the ESC19 Strain

**FIGURE 2:**
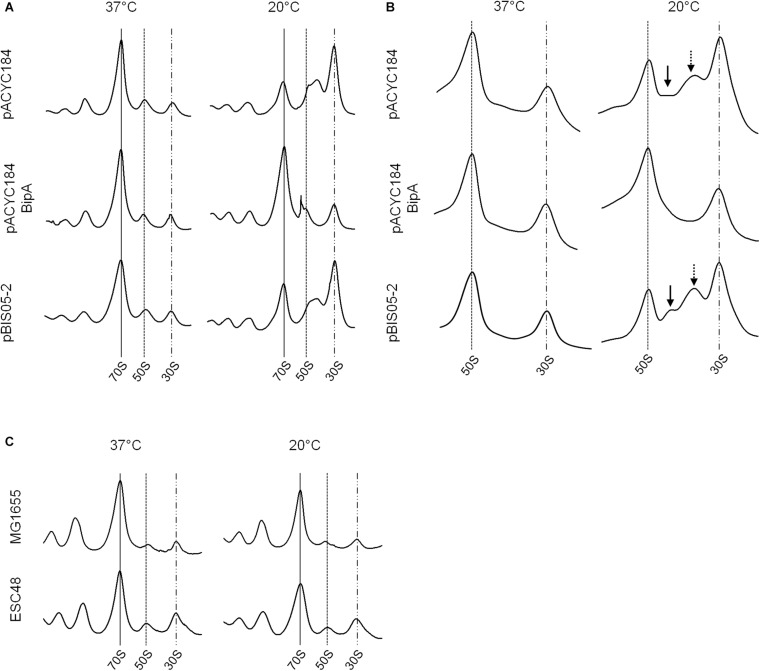
Ribosomal abnormality retained in the suppressor strain. Polysome profiles **(A)** and subunit profiles **(B)** of ESC19 cells harboring pACYC184, pACYC184BipA, and pBIS05-2. Transformants were grown at 37°C or 20°C in LB medium containing chloramphenicol until they reached an OD_600_ of ∼0.6. Then, cell lysates were prepared and analyzed as described in section “Materials and Methods.” Arrows indicate abnormal ribosomal particles. **(C)** Analyses of polysome profiles of the MG1655 and ESC48 strains. The polysomes were analyzed using the lysate of cells grown at 37°C or 20°C in LB medium until the exponential phase as in **(A)**.

As discussed above, BipA may play global regulatory roles in capsule formation, motility, and pathogenicity in cells. Among these, the most apparent phenotype conferred by the *bipA-*deletion is cold-sensitivity caused by failure of 50S ribosomal subunit assembly (Choi and Hwang, 2018). We have shown that the exogenous expression of *rplT* restored the growth of the ESC19 strain at low temperature by restoring aberrant 50S ribosomal subunit assembly (Choi et al., 2019). Thus, to investigate whether the ribosomal defects of the ESC19 strain could be suppressed by overexpression of *yebC*, the polysomes and ribosomal subunits of the suppressed cells were analyzed by sucrose density gradient sedimentation experiments. ESC19 cells harboring pACYC184, pACYC184BipA, and pBIS05-2 were cultivated at 37°C or 20°C until mid-exponential phase and collected by centrifugation. Then, the cell pellets were subjected to sucrose density gradient sedimentation as described in section “Materials and Methods.” As expected, ESC19 cells harboring any of the plasmids exerted normal polysome and subunit profiles without any aberrant or accumulated particles at 37°C (left panels in Figures 2A,B). However, at low temperature, ESC19 cells harboring pACYC184 accumulated a significant level of free 30S ribosomal subunits in the polysome profile. Notably, the peak of 50S ribosomal subunits was almost half of that of 30S ribosomal subunits, and shoulder peak appeared to the right of 50S ribosomal subunits (top right panel in Figure 2A). Consistently, two abnormal particles appeared between the 50S and 30S ribosomal subunit peaks with a concomitant reduction of 50S ribosomal subunits in the subunit profile results for this strain (top right panel in Figure 2B). These results suggest that the absence of BipA at low temperature caused accumulation of unprocessed precursors and/or destabilized 50S ribosomal subunits. ESC19 cells expressing *bipA* showed a normal accumulation of 50S and 30S ribosomal subunit particles in polysomes at both temperatures (Figure 2A, center). Likewise, unprocessed precursors and/or destabilized 50S ribosomal subunits did not appear between 50S and 30S ribosomal subunit peaks at either temperature (Figure 2B, center), indicating complementation of pACYC184BipA. Interestingly, ESC19 cells transformed with pBIS05-2 exerted very similar polysome profile to those of ESC19 cells transformed with pACYC184 at 20°C (bottom right panel in Figure 2A). Consistently, abnormal 50S ribosomal subunit particles appeared between 50S and 30S peaks in the subunit profiles at 20°C (bottom right panel in Figure 2B). These results suggest that the *yebC* suppressor gene did not contribute significantly to 50S ribosomal subunit biogenesis, although it alleviated cold-sensitivity. Notably, both normal and abnormal 50S subunit particles overlapped at similar positions in polysome profiles because of a high sucrose gradient concentration and short sedimentation distance (right panel in Figure 2A).

In order to exclude a supportive role of YebC in 50S ribosome biosynthesis, the polysome profiles of wild-type (MG1655) and *yebC*-deleted (ESC48) strains were explored. The *yebC* mutant showed no significant ribosomal defects compared to wild-type at either temperature, suggesting that the function of YebC is not associated with ribosome assembly under the given conditions (Figure 2C). This raises the question of whether the cold-sensitivity of the *bipA*-deleted mutant is not solely due to ribosomal defects.

### Colony Morphology Without Capsule in Suppressed Cells

**FIGURE 3:**
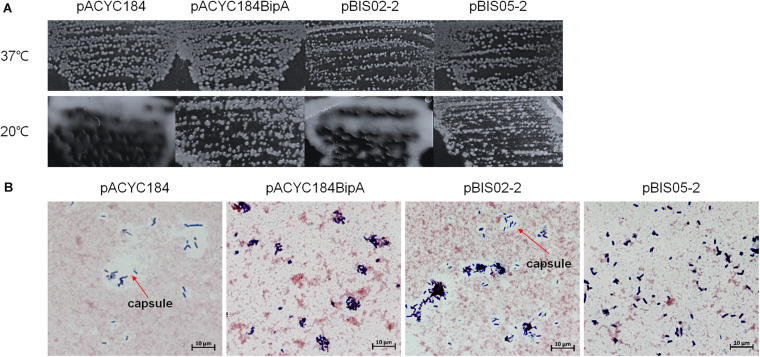
Colony morphologies of the ESC19 transformants. **(A)** ESC19 cells transformed with pACYC184, pACYC184BipA, pBIS02-2, or pBIS05-2 were grown on LB agar plates supplemented with the appropriate antibiotics overnight at 37°C or 4 days at 20°C. The suppressor clone (pBIS02-2) expressing r-protein L20 was tested as a mucoid suppressor. **(B)** Capsule staining by crystal violet and Congo red. Colonies from LB plates incubated at 20°C for 4 days were stained and observed as described in section “Materials and Methods.” Arrows indicate capsule.

Interestingly, when we initially confirmed the suppression activities of pBIS02 (a library clone expressing r-protein L20) and pBIS05, we noticed that transformants with pBIS05 were non-mucoid at low temperature unlike those with pBIS02. Therefore, to elucidate a possible role of YebC in suppression at low temperature, we observed the colony morphologies of the suppressed cells. First, we confirmed capsule production in ESC19 at low temperature as shown in Supplementary Figure S2A, and a clear zone around ESC19 cells was visualized using crystal violet and Congo red staining methods (Supplementary Figure S2B). Next, pACYC184, pACYC184BipA, pBIS02-2 (a subclone of pBIS02), or pBIS05-2 was transformed to the ESC19 strain, and the transformants were streaked onto LB agar plates containing chloramphenicol and kanamycin followed by incubation at 37°C or 20°C. As shown in Figure 3A, all transformants were non-mucoid at 37°C. However, at low temperature, the ESC19 strain with pACYC184 showed a substantially mucoid phenotype, which was reversed to non-mucoid when pACYC184BipA was introduced. Likewise, transformants with pBIS05-2 showed the same morphological phenotype at 20°C without being mucoid. Notably, pBIS02-2 and pACYC184BipA_N128D_ did not restore the mucoid phenotype (Figure 3A and Supplementary Figure S3A).

Colonies of these transformants were stained with crystal violet and Congo red to microscopically observe individual cells as described in section “Materials and Methods.” In Figure 3B, a large clear zone was observed surrounding ESC19 cells harboring pACYC184, which was complemented by expression of *bipA* from pACYC184BipA. However, the ESC19 strain harboring pBIS05-2 did not form the clear zone, which is formed by excessive production of exopolysaccharide. These results suggest that the deletion of *bipA* at low temperature led to capsule overproduction and that overexpressed YebC in ESC19 cells may repress production of capsule. Consistently, ESC19 transformants with pBIS02-2 had a clear exopolysaccharide layer around them, indicating that overexpressed L20 in ESC19 cells was unable to repress capsule synthesis. The ESC48 strain did not produce excessive capsular exopolysaccharide at 20°C (Supplementary Figure S2). These results imply that the function of BipA in capsule production repression at low temperature may prevail over that of YebC.

### Repression of the Operon by YebC

To investigate the role of YebC in suppressing capsule production, transcriptional changes in genes that are involved in capsule synthesis were analyzed using qRT-PCR. We chose six genes in the *cps* operon, a gene cluster functioning in CA biosynthesis (Stevenson et al., 1996; Rahn et al., 1999). The *gmd* and *ugd* genes are involved in CA precursor synthesis (Stevenson et al., 1996; Sturla et al., 1997), and *wcaA* and *wcaK* encode CA modification enzymes (Scott et al., 2019). WcaD polymerizes CA units, and Wzc exports colonic acid across the inner membrane (Stevenson et al., 1996; Paulsen et al., 1997).

**FIGURE 4:**
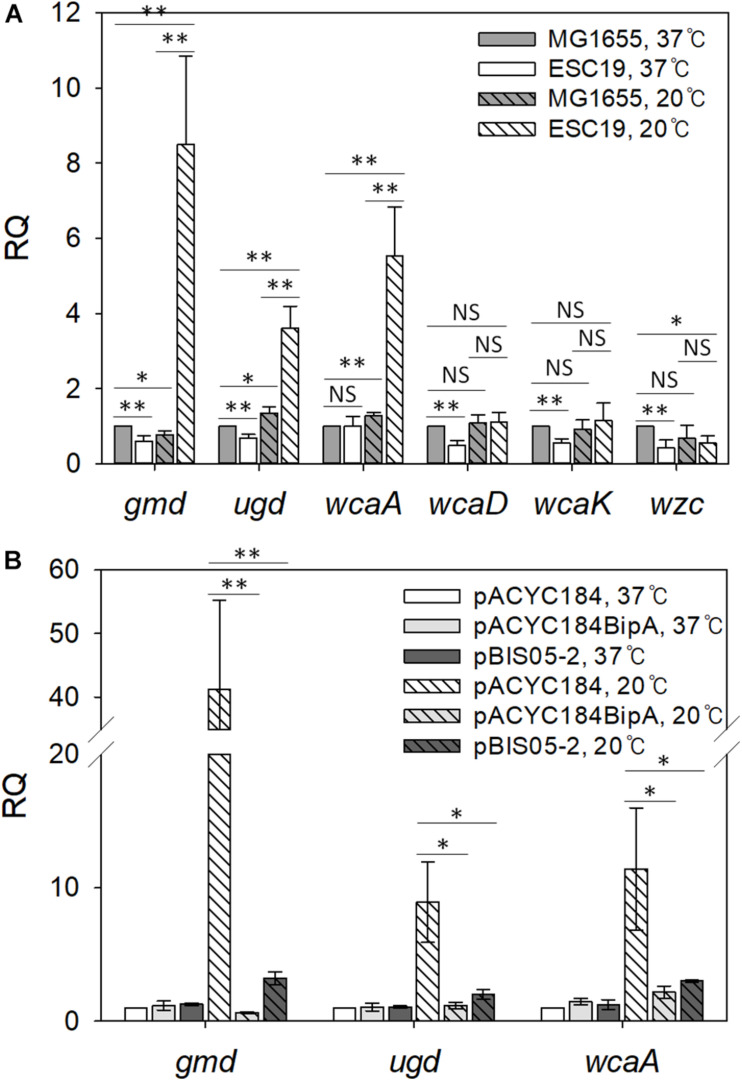
Analysis of transcript levels of genes involved in colanic acid synthesis. **(A)** Quantification of transcript levels of the *cps* genes in MG1655 and ESC19 strains at 37°C or 20°C. Cells were grown on LB agar plates at 37°C for 18 h or at 20°C for 5–6 days, and total RNAs were extracted and analyzed using qRT-PCR for *gmd*, *ugd*, *wcaA*, *wcaD*, *wcaK*, and *wzc* transcripts. The expression levels were calculated relative to the expression level of MG1655 grown at 37°C. **(B)** qRT-PCR analysis of *gmd*, *ugd*, and *wcaA* in ESC19 transformants with pACYC184, pACYC184BipA, or pBIS05-2 at 37°C or 20°C. The expression levels were calculated relative to the expression level of ESC19 with pACYC184 grown at 37°C. The expression levels were normalized to that of the endogenous control gene *gapA* (glyceraldehyde-3-phosphate dehydrogenase A). Error bars denote SD. NS, non-significant; ^∗^*p* < 0.05; ^∗∗^*p* < 0.01.

First, we compared the mRNA levels of those genes in strains MG1655 and ESC19 grown at 37°C or 20°C. Cells grown on LB agar plates were collected by scraping, total RNAs were extracted from the collected cells, and qRT-PCR analysis was conducted. As shown in Figure 4A, both strains grown at 37°C did not have significant changes in transcript levels of any of the six genes. However, at 20°C, the transcript levels of *gmd* (11.1-fold), *ugd* (2.7-fold), and *wcaA* (4.3-fold) were markedly higher in the ESC19 strain than in MG1655, demonstrating capsule overproduction in ESC19 at 20°C.

Next, to examine whether YebC could repress the expression of *gmd*, *ugd*, and *wca* genes, we carried out the same experiment using the suppressed strain. As we expected, in the ESC19 cells with pBIS05-2 at 20°C, RQ values for *gmd*, *ugd*, and *wcaA* transcripts decreased to 3.2, 2.0, and 3.8, respectively, indicating similar expression levels to the complemented strain (Figure 4B). At 37°C, there were no significant differences in the expression levels of these three genes among transformants harboring each plasmid. These results suggest that overexpressed YebC led to a reduction in transcript levels of *gmd, ugd*, and *wcaA*, and subsequently restored capsule production in the ESC19 strain at 20°C. Considering that Gmd, Ugd, and WcaA precede WcaD, WcaK, and Wzc in CA biosynthesis (Ranjit and Young, 2016; Scott et al., 2019), it is likely that YebC may be involved in regulation of earlier steps of CA synthesis at low temperature.

### Interrupted LPS Core Biosynthesis in bipA-Deleted Cells

**FIGURE 5:**
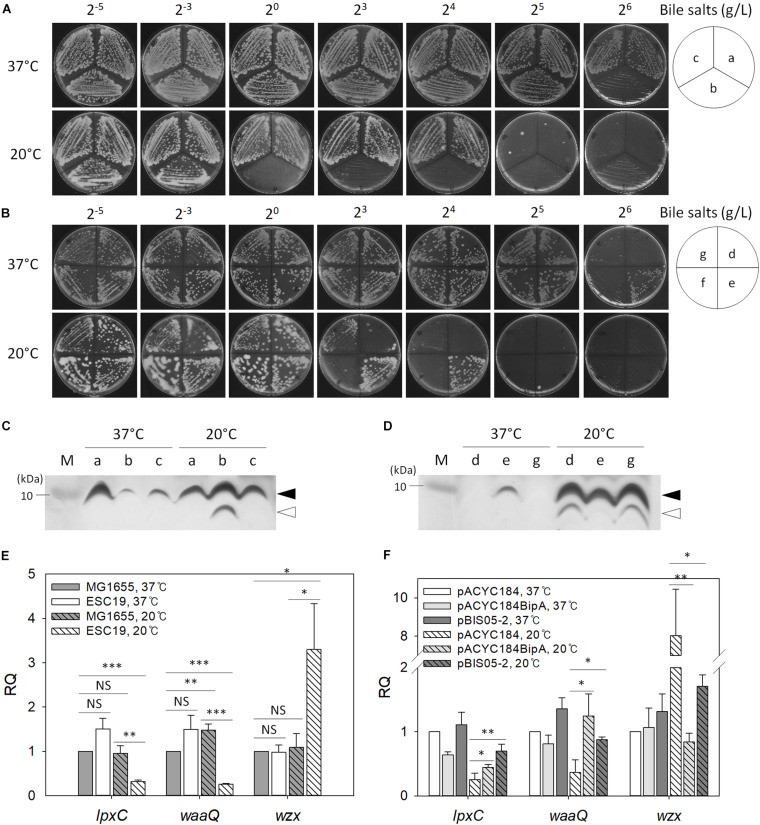
The effect of *bipA*-deletion on LPS biosynthesis. Overnight cultures of the MG1655, ESC19, and ESC48 strains **(A)** or the ESC19 transformants with pACYC184, pACYC184BipA, pBIS02-2, or pBIS05-2 **(B)** were diluted with LB medium to an OD_600_ of 0.2. Then, 3 μl of the diluted cultures were streaked onto LB plates supplemented with different concentrations of bile salts as presented above, after which the plates were incubated at 37°C or 20°C. The media for transformants contained chloramphenicol and kanamycin. **(a)**, MG1655; **(b)**, ESC19; **(c)**, ESC48; **(d)**, ESC19 with pACYC184; **(e)**, ESC19 with pACYC184BipA; **(f)**, ESC19 with pBIS02-2, and **(g)**, ESC19 with pBIS05-2. **(C,D)** Tricine SDS-PAGE analysis of LPS. LPS was extracted as described in section “Materials and Methods” and subjected to 12.5% Tricine-polyacrylamide gel electrophoresis followed by silver staining. M, PageRuler^TM^ Unstained Protein Ladder (Thermo Fisher Scientific^TM^). Alphabetical labels are the same as in **(A,B)**. Filled and open arrows represent mature and precursor LPS core, respectively. **(E,F)** Relative quantification of mRNA levels of genes involved in LPS biosynthesis. Total RNA was extracted and subjected to qRT-PCR analysis. The relative quantities of *lpxC*, *waaQ*, and *wzx* were normalized to that of the endogenous control gene *gapA*. Error bars denote the SD using three experimental replicates. NS, non-significant; ^∗^*p* < 0.05; ^∗∗^*p* < 0.01; ^∗∗∗^*p* < 0.001.

Recently, it was reported that CA synthesis is activated in response to a defect in the core oligosaccharide of LPS through the regulator of capsule synthesis (Rcs) phosphorelay system, resulting in secretion of large amounts of exopolysaccharide and mucoid colony morphology (Ren et al., 2016). Furthermore, as mentioned above, BipA seems to be functionally associated with LPS core synthesis (Moller et al., 2003). Therefore, we examined the sensitivity of the suppressor strains to bile salts, to which *E. coli* cells with defective LPS core are known to be hypersensitive (Picken and Beacham, 1977). Overnight cultures of MG1655, ESC19, or ESC48 strains were streaked onto LB agar plates supplemented with 2^–5^ to 2^6^ g/L bile salts and incubated at 37°C or 20°C. As shown in Figure 5A, the ESC19 strain did not form colonies on the LB plate containing 2^6^ g/L bile salts, whereas MG1655 and ESC48 strains grew normally at 37°C in the presence of bile salts at concentrations up to 2^6^ g/L. In contrast, the growth of MG1655 and ESC48 strains were inhibited by 2^5^ g/L bile salts at 20°C. Interestingly, ESC19 cells did not form colonies at a much lower concentration of bile salts (2^0^ g/L). The hypersensitivity of ESC19 to bile salts at low temperature implies that *E. coli* may go through changes in composition or structure of LPS core at 20°C and that BipA is functionally implicated in LPS synthesis at 20°C. Next, the suppressor clones were transformed into ESC19 cells to examine whether YebC could recover the sensitivity of ESC19 cells to bile salts at 20°C. Each transformant was streaked on LB plates containing bile salts and incubated at 37°C or 20°C. The complemented ESC19 strain with pACYC184BipA grew well at 37°C on LB plates containing bile salts without any abnormality, while the growth of the other ESC19 transformants were inhibited by 2^6^ g/L bile salts (Figure 5B). However, at 20°C, the ESC19 transformant with pACYC184 became sensitive at ≥2^3^ g/L of bile salts. Notably, ESC19 cells with pBIS05-2 were insensitive to 2^3^ g/L bile salts. The growth of ESC19 cells harboring pACYC184 was inhibited at this concentration of bile salts, suggesting that partial suppression by YebC may be mediated by the recovery of LPS biosynthesis.

Next, to investigate any LPS core defect in the ESC19 strain, we analyzed the LPS extracted from the various strains. The LPS isolated from MG1655, ESC19, and ESC48 strains were examined using Tricine SDS-PAGE followed by silver staining. As shown in Figure 5C, ESC19 cells showed a normal LPS core, although accumulation was reduced at 37°C. Interestingly, LPS of the ESC19 strain exhibited increased amounts of mature LPS core with a concomitant accumulation of LPS core precursor at 20°C. The upper LPS core band was almost undetectable in the ESC19 transformants with pACYC184 or pBIS05-2, probably because the very small amount of LPS core in ESC19 cells was rapidly consumed as a substrate for O-antigen polymerization at 37°C. However, at 20°C, cells harboring pACYC184 or pBIS05-2 accumulated LPS core precursor, which was not observed in ESC19 with pACYC184BipA. Our results suggest that maturation of LPS core at low temperature is affected by the deletion of *bipA*, eventually making this mutant sensitive to bile salts.

To further verify the impairment of LPS core synthesis, qRT-PCR was carried out targeting genes involved in lipid A synthesis (*lpxC*), core oligosaccharide synthesis (*waaQ*), and polysaccharide synthesis (*wzx*) (Anderson et al., 1985, 1988, 1993; Liu et al., 1996; Yethon et al., 1998). As shown in Figure 5E, significant changes were not observed with any of the three transcripts in both strains at 37°C, while the ESC19 strain showed substantial reductions in the RQ values for *lpxC* (0.32) and *waaQ* (0.26) at 20°C. In contrast, *wzx* transcript levels increased 3.0-fold compared to the MG1655 grown at 20°C, suggesting that the initial steps of LPS biosynthesis may be hampered in ESC19 cells at low temperature.

Interestingly, when ESC19 cells with pBIS05-2 were grown at 20°C, these transcriptional changes were reversed. As shown in Figure 5F, qRT-PCR analysis revealed that the expression levels of *lpxC* and *waaQ* were significantly reduced (4.0- and 2.7-fold, respectively) in ESC19 cells with pACYC184 grown at 20°C compared to those grown at 37°C. Transcript levels of *lpxC* and *waaQ* were restored by overexpression of *yebC* to similar levels as complemented ESC19 cells. These results suggest that partial restoration of bile salt sensitivity by YebC resulted from modulation of the transcriptional profile of genes involved in LPS synthesis.

### The Effect of YebC on Biofilm Formation by the ESC19 Strain

**FIGURE 6:**
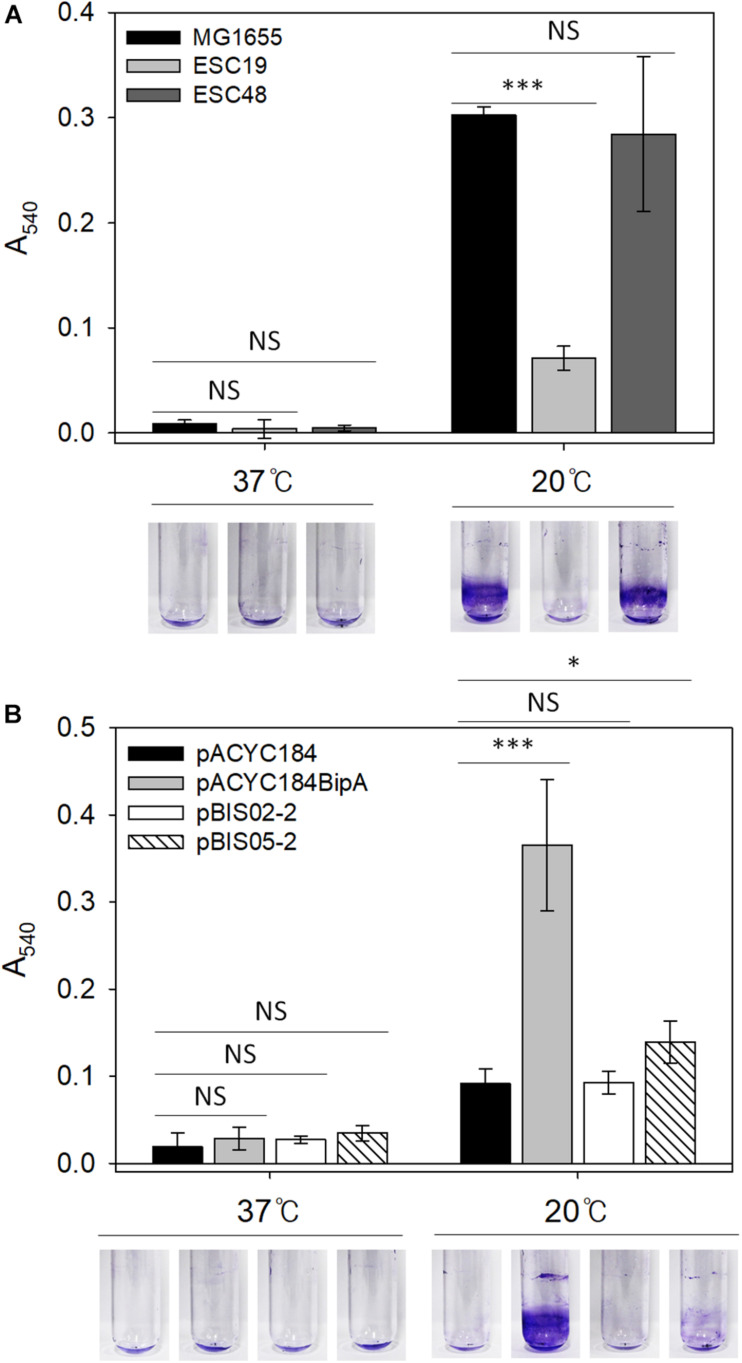
Biofilm formation assay of MG1655, ESC19, and ESC48 strains **(A)** and ESC19 transformants with pACYC184, pACYC184BipA, pBIS02-2, or pBIS05-2 **(B)**. Cells were grown in 2.5 ml of LB broth with appropriate antibiotics at 37°C for 24 h or 20°C for 48 h. Then, biofilm biomass on inner surface of glass tube was stained with 0.1% crystal violet and dissolved in 95% ethanol. The absorbance of the resulting dissolved dye was measured at a wavelength of 540 nm. A representative photograph is shown from three independent experiments. Error bars represent SD using three replicates. NS, non-significant; ^∗^*p* < 0.05; ^∗∗∗^*p* < 0.001.

Mutations in LPS synthesis have been shown to affect the ability of *E. coli* to adhere to abiotic surfaces, leading to significantly reduced biofilm formation (Genevaux et al., 1999). In the case of CA capsule, it has been known to play an inhibitory role in biofilm formation by shielding bacterial surface adhesin and releasing capsular polysaccharide (Roberts, 1996; Whitfield, 2006). Therefore, weakened cell-surface contacts and reduced cell-cell interactions can antagonize biofilm maturation (Beloin et al., 2008). Lastly, since the deletion of *bipA* was also reported to impair biofilm formation in *P. aeruginosa* (Overhage et al., 2007; Neidig et al., 2013), we investigated whether the reduced capsule production and restoration of LPS core maturation in suppressed cells can affect biofilm formation under the given conditions. To address this, biofilms were quantified using the crystal violet staining method. First, to assess the effect of *bipA* or *yebC* deletion on biofilm-forming ability, the strains MG1655, ESC19, and ESC48 were grown in LB medium at 37°C for 18 h or 20°C for 48 h. The biofilms formed on the inner surface of glass tubes were quantified as described in section “Materials and Methods.” As shown in Figure 6A, the MG1655 strain cultivated at 20°C showed a noticeable increase in biofilm formation (38-fold) compared to cells grown at 37°C and to ESC48 cells. Interestingly, the amount of biofilm produced was greatly reduced in the ESC19 strain (4.3-fold) compared to MG1655 at 20°C.

Next, to examine whether YebC could restore impaired biofilm-forming ability in the ESC19 strain, the biofilms produced by ESC19 transformants with pACYC184, pACYC184BipA, pBIS02-2, or pBIS05-2 were quantified. The biofilms produced by the complemented ESC19 cells harboring pACYC184BipA were significantly increased at 20°C compared to those of ESC19 cells with pACYC184 (Figure 6B). ESC19 cells expressing YebC showed slightly increased biofilm production, suggesting that expression of *yebC* partially restored the biofilm-forming ability of ESC19 at 20°C. The defective biofilm production of pBIS02-2 was not recovered. These findings suggest that biofilm formation was prevented by overproduced capsule in ESC19 cells at 20°C and that overexpressed YebC resulted in partial restoration of biofilm production by repressing capsule synthesis.

## Discussion

In this study, we have shown that the global regulator BipA is functionally associated with ribosome biosynthesis, capsule production, LPS core synthesis, and biofilm formation. Among the pleiotropic phenotypes caused by *bipA*-deletion, the most conspicuous was defective ribosome assembly at low temperature, although BipA was not essential for this function at 37°C (Choi and Hwang, 2018). Defective rRNA processing and ribosome assembly causes many ribosome assembly factor mutants to be cold-sensitive (Connolly and Culver, 2009). Interestingly, overexpression of *yebC* in the *bipA* mutant did not rescue ribosomal abnormalities, however, confers an ability to grow at low temperature (Figure 2). This prompts us to speculate that BipA is involved in other cellular functions as well as ribosome biogenesis. As a ribosome-associated GTPase, BipA shares several characteristic features with other trGTPases, such as LepA and EF-G (Ero et al., 2016). The primary and tertiary structures of BipA are highly similar with those of LepA and EF-G, and the enzymatic behaviors of BipA are the same as these proteins regarding GTP-dependent ribosome binding and ribosome-induced GTP hydrolysis. Furthermore, the ribosomal binding sites of these three GTPases overlap (Ero et al., 2016).

This leads us to assume that BipA is very likely to function as a translational GTPase. From this point of view, we can hypothesize three possibilities regarding the roles of BipA. First, BipA may engage in assembly of subgroups of complete ribosomes at low temperature. The *de novo* synthesis of ribosomes is regulated in response to nutritional starvation and various stress conditions, and recent reports demonstrate that in *E. coli*, r-proteins L31 and L36 are encoded by *rpmE/ykgM* (or *rpmE2*) and *rpmJ/ykgO* (or *rpmJ2*), respectively (Lilleorg et al., 2019; Ghulam et al., 2020). In *E. coli* K-12, there are seven redundant rRNA genes having heterogeneous nucleotide sequences, and the most variable regions are located at helices 63 and 98 (Anton et al., 1999). Furthermore, the promoters of these rDNA operons were differentially responsive to various stimuli, causing an accumulation of distinct ribosome species under various environmental conditions (Condon et al., 1992; Maeda et al., 2015; Kurylo et al., 2018). Therefore, expression of rRNAs or r-protein paralogs ultimately forms a dynamic population of ribosomes with heterogeneous protein composition depending on growth conditions. Ribosomes act as thermosensors (Vanbogelen and Neidhardt, 1990), and upon cold-shock, assembly factors such as CsdA and RbfA are promptly induced, indicating that remodeling by rRNA folding and r-protein incorporation may be undertaken to facilitate proper ribosome biosynthesis (Jones and Inouye, 1996; Jones et al., 1996). During this process, BipA may recognize those heterogeneous ribosome populations to promote effective ribosome assembly at low temperature. Recently, these divergent ribosomes were revealed to contribute to differential translation of a body of transcripts in *Vibrio vulnificus* (Song et al., 2019).

Second, whole transcription profiles vary between 37 and 20°C, especially during the acclimation phase (Phadtare et al., 1999), and the effective translation of cold-shock related genes is required for cells to adapt to low temperature. The absence of BipA in cold-exposed cells may cause a cold-sensitive phenotype with deficient expression of a factor(s) that are required for normal growth under cold-stress conditions. The cold-shock inducible protein, initiation factor IF3, was shown to preferentially translate cold-shock mRNAs (Giuliodori et al., 2004; Bruijn, 2016). Therefore, it is possible that BipA itself may act as a translational factor with bias.

Lastly, in concert with the two hypothetical roles discussed above, BipA may regulate the transcription of capsule or LPS-related genes either directly or indirectly. As mentioned earlier, premature transcription terminator sequences were inserted into *waaQ*, a promoter-adjacent gene in the *waa* operon, which shuts down the transcription of the entire operon. However, an additional *bipA* mutation in the *waaQ* mutant relieved the failure in LPS core synthesis by an uncharacterized mechanism, implying that genes downstream of *waaQ* are somehow transcribed by mutant BipA (Moller et al., 2003).

BPI interacts with the lipid A moiety of LPS and competitively replaces Ca^2+^ and Mg^2+^ divalent metal ions, which chelate the negative charge of phosphates in LPS. This interaction consequently perturbs the normal arrangement of LPS molecules, resulting in membrane rupture and eventually cytotoxicity (Levy, 2000). Upon exposure to this antimicrobial protein, *Salmonella typhimurium* induces the expression of *bipA* (Qi et al., 1995). Thus, it is likely that BipA may sense perturbations in the outer membrane and modulate the expression of capsule and/or LPS-related genes. We speculate that in the absence of BipA at low temperature, expression of LPS core production genes may be disturbed, eventually damaging outer membrane integrity. In addition to LPS-triggered damage, cold-shock stress induces a decrease in membrane fluidity, an increase in permeability, and a change in fatty acid composition of lipid A (Carty et al., 1999; Cao-Hoang et al., 2008, 2010). Mutant r-protein L6 also affects membrane stability (Bosl and Bock, 1981). It is noteworthy that the *bipA* mutant accumulates L6-deficient 50S ribosomal subunits at low temperature (Choi and Hwang, 2018). Thus, under the combinatorial effects of injuries or alternations of membrane and LPS, the Rcs phosphorelay system is likely to stimulate capsule production. In *E. coli*, CA capsule is synthesized and tightly controlled by a signal transduction system governed by the Rcs phosphorelay system (Trisler and Gottesman, 1984; Stout and Gottesman, 1990). Upon receiving signals, an outer membrane-integrated lipoprotein, RcsF, interacts with an inner membrane protein, IgaA and this interaction activates the downstream RcsB-RcsC two-component system (Cho et al., 2014). This activation phosphorylates the cytosolic domain of RcsC, a transmembrane sensor histidine kinase. Then, the phosphate group is transferred to RcsD, an intermediate inner membrane phosphorelay protein, which is eventually transferred to the effector protein, RcsB (Takeda et al., 2001; Hagiwara et al., 2003; Majdalani and Gottesman, 2005). Phosphorylated RcsB functions as a transcriptional activator for the expression of the *cps* operon for capsule formation (Stout and Gottesman, 1990). RcsB must form a heterodimer complex with another positive regulator, RcsA, in order to regulate the transcription of the *cps* operon (Stout et al., 1991). Furthermore, expression of *rcsA* is negatively regulated by H-NS and CRP, consequently repressing capsule production (Sledjeski and Gottesman, 1995; Lin et al., 2013). Intriguingly, however, the *rcsF*-*bipA* double deletion mutant was able to produce capsule at low temperature (Supplementary Figure S5). This may be inferred by the facts that RcsB can respond to another signal and that the repressed expression of *igaA* in ESC19 cells triggers the Rcs system, consequently producing exopolysaccharide capsule (Potrykus and Wegrzyn, 2004).

Unlike capsule production, the activated Rcs system shuts down the expression of *flhDC* for flagella production, leading to immobility (Francez-Charlot et al., 2003). We have confirmed that the ESC19 strain showed almost no motility at 20°C and that pBIS02-2 and pBIS05-2 did not contribute to cellular motility (Supplementary Figure S4), suggesting that YebC functions in capsule and LPS core synthesis but not motility.

Here, we demonstrated that overexpression of *yebC* ameliorated defects in growth and capsule synthesis of ESC19 at low temperature (Figures 1, 3). In addition, suppressed cells were insensitive to bile salts and displayed increased mRNA levels of *lpxC* and *waaQ* (Figure 5), suggesting that the role of YebC in the suppression is confined to the regulation of capsule production and LPS core synthesis. Thus, based on our findings and earlier reports on DNA binding activity of YebC, it is likely that YebC in the *bipA* mutant specifically represses the transcription of capsule and LPS core synthesis genes. Currently, we are carrying out SELEX (Systematic evolution of ligands by exponential enrichment) experiments to isolate target gene(s) that YebC may bind and modulate. This will help us to gain further insights into the physiological function of YebC. The most intriguing finding is that BipA controls the LPS core biosynthesis at low temperature, which may ultimately explain the pleiotropic phenotypes of *bipA-*deletion. This present study is the first report that investigates the role of YebC in *E. coli* and demonstrates the relationship of YebC with capsule and LPS core synthesis. Taken together, our results will provide a better understanding of the role of BipA in the cold-stress response, LPS core synthesis, and capsule synthesis.

## Data Availability Statement

The original contributions presented in the study are included in the article/Supplementary Material, further inquiries can be directed to the corresponding author.

## Author Contributions

EC designed the study, executed the experiments, analyzed the data, and wrote the manuscript. HJ and CO assisted the experiments. JH supervised EC and HJ. All authors discussed the results and approved the final manuscript.

## Conflict of Interest

The authors declare that the research was conducted in the absence of any commercial or financial relationships that could be construed as a potential conflict of interest.
