# Supplementary material for: Elucidation of a Novel Role of YebC in Surface Polysaccharides Regulation of Escherichia coli bipA-Deletion
Source: Front Microbiol. 2020 Nov 9;11:597515. doi: 10.3389/fmicb.2020.597515 (PMC7682190; doi:10.3389/fmicb.2020.597515)
Supplement: Supplementary file 1 [file Data_Sheet_1.docx]

Supplementary Material

**Supplementary Table S1. Primers used in this study**

| **Primers** | **Sequences (5’ to 3’)** | **Source or reference** |
| --- | --- | --- |
| BIS05-F-*Bam*HI | GAGGATCCTGGATCGTACTGCCGC | This study |
| BIS05-1-R-*Bam*HI | AAGGATCCAAAAATATCTCCAGATA | This study |
| BIS05-2-R-*Bam*HI | TAGGATCCCACGCGTCTCCGTTTTG | This study |
| BIS05-3-R-*Bam*HI | AAGGATCCCTTAACGCAGTCGCCC | This study |
| *yebC*-stop-F | TCTGGAGATATTTTTTAGGCAGGTCATAGTAA | This study |
| *yebC*-stop-R | TTACTATGACCTGCCTAAAAAATATCTCCAGA | This study |
| *rcsF*-100-U | CAAAGTGTTTAACGGCGGAG | This study |
| *rcsF*-100-D | TGGTTGTAGGGAGCAATGAG | This study |
| *gapA*-RT-5 | AAGTTGGTGTTGACGTTGTCGC | (Navasa et al., 2011) |
| *gapA*-RT-3 | AGCGCCTTTAACGAACATCG | (Navasa et al., 2011) |
| *gmd*-RT-5 | TTGAAGAGAAGGGCATTGTG | This study |
| *gmd*-RT-3 | GAGATTTCAGCAGAGAGTGT | This study |
| *ugd*-RT-5 | TATGGTGGTTATTGTCTGCC | This study |
| *ugd*-RT-3 | GATCACTTCAACACCTTTCG | This study |
| *wcaA*-RT-5 | TTTGTACGCTAACGACTACG | This study |
| *wcaA*-RT-3 | CTCTTCTACTTTCCACGGTT | This study |
| *wcaD* -RT-5 | TTCTTTTATCTGGTGGTCGG | This study |
| *wcaD* -RT-3 | GCATCTGTTTTAGGCGTTTT | This study |
| *wcaK* -RT-5 | CCTGCAAATGAAACAACACA | This study |
| *wcaK* -RT-3 | GCAAAGTGCATGTTCAAACT | This study |
| *wzc*-RT-5 | AAAAGCCAAACTTAACGGTC | This study |
| *wzc*-RT-3 | ATAGAAAGCATCAGGCCAAG | This study |
| *lpxC*-RT-5 | ATGGTTTTTCGCTGGATTTC | This study |
| *lpxC*-RT-3 | CTTCGTTCAGTACGCGATAA | This study |
| *waaQ*-RT-5 | ATTTCTACACCGACACAACA | This study |
| *waaQ*-RT-3 | ATCTCATCTACACAAGCGAG | This study |
| *wzx*-RT-5 | TTCACTTGTACGGTGGTTAG | This study |
| *wzx*-RT-3 | ATAGTTGATGATGCTGTCCG | This study |

**Supplementary Figure S1.** Suppressive acitivity of pBIS05 and its derivative. Growth of ESC19 transformants with pAYC184, pACYC184BipA, pBIS05, or pBIS05-stop was measured as described in Fig. 1. Experiments were performed in two independent replicates and error bars represent S.D.

**A**


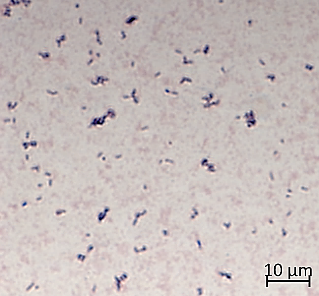

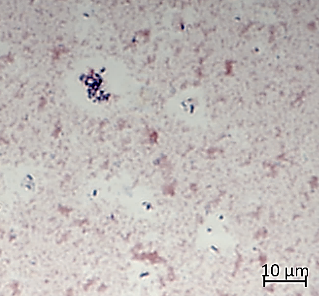

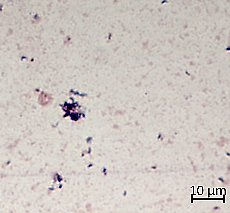


MG1655

ESC48

ESC19

**capsule**

MG1655

ESC19

ESC48

**B**


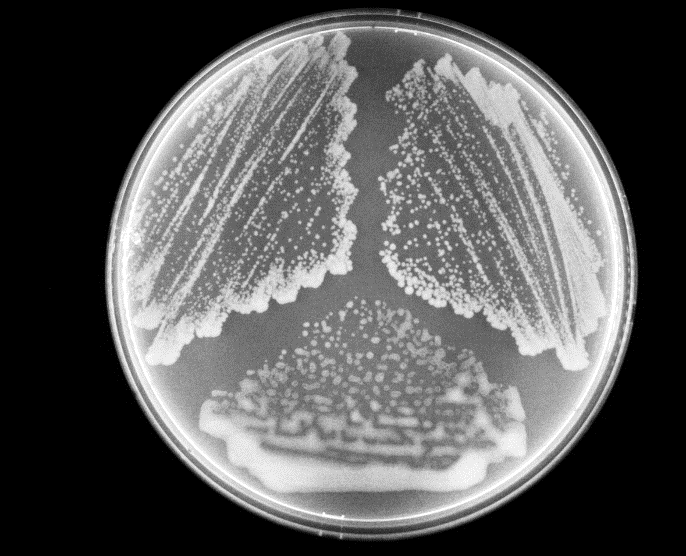

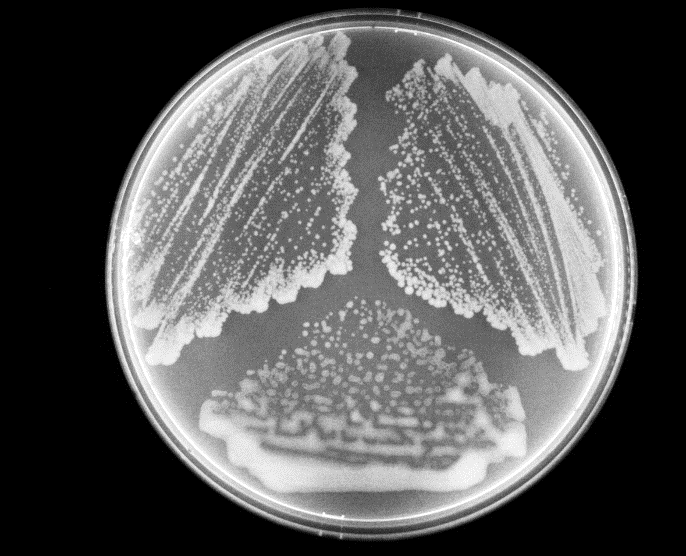

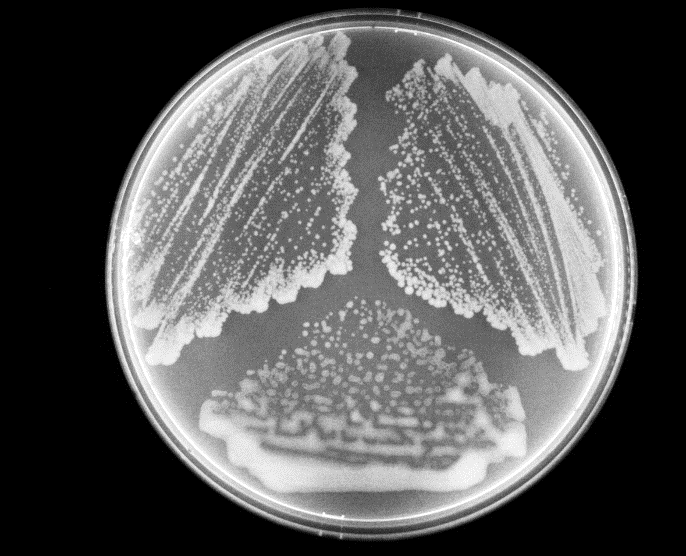


**Supplementary Figure S2.** Colony morphologies of the strains MG1655, ESC19, or ESC48. **(A)** Each strain was grown overnight on LB agar plate for 4 days at 20°C. **(B)** Capsule staining by Crystal violet and Congo red. Colonies from LB plates incubated at 20°C for 4 days were stained and observed as described in Materials and Methods. Arrows indicate capsule.


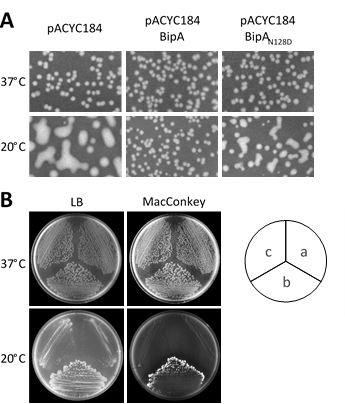


**Supplementary Figure S3.** Effect of GTPase activity of BipA on capsule production and LPS biosynthesis. **(A)** Colony morphologies of ESC19 transformants harboring pACYC184, pACYC184BipA, or pACYC184BipA_N128D_. 75 μl of diluted overnight culture was spread on to LB agar plates containing chloramphenicol and kanamycin, then the plates were incubated at 37°C or 20°C. **(B)** Growth of ESC19 transformants on LB or MacConkey agar plates. The ESC19 cells shown in **(A)** were incubated overnight in LB medium containing chloramphenicol and kanamycin at 37°C. 3 μl of diluted overnight culture was streaked on LB or MacConkey agar plates containing the same antibiotics. a; pACYC184, b; pACYC184BipA, c; pACYC184BipA_N128D._

**A**

**B**


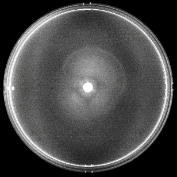

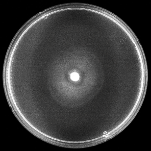

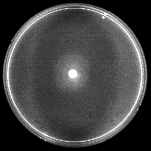


37°C

20°C

pACYC184

pACYC184

BipA

pBIS02-2

pBIS05-2


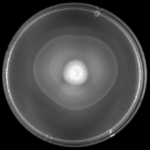

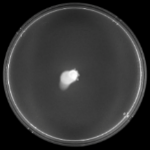

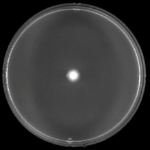

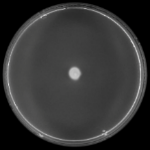


**D**

**C**

MG1655

ESC19

37°C

20°C

ESC48


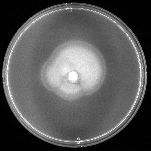

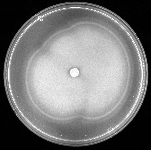

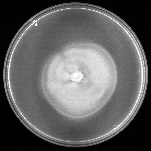

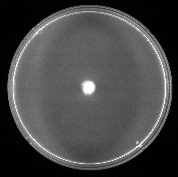

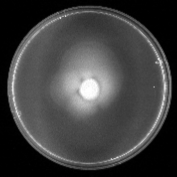

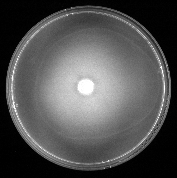

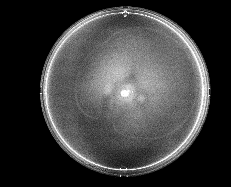


**Supplementary Figure S4.** Swimming motility assay. **(A), (B)** Motilities of the strains MG1655, ESC19 and ESC48 at 37°C or 20°C on swimming LB agar plates (0.3% agar). The swimming speed was calculated by dividing migration distance by incubation time. The results are representative of three identical individual assays. Representative result with the strains MG1655 and ESC48 after 16 h at 37°C or 7 days at 20°C is shown in **(A)**. **(C), (D)** Motilities of ESC19 cells transformed with pACYC184, pACYC184BipA, pBIS02-2, or pBIS05-2. The assay was performed triplicate as described above. The representative result of each assay is shown in **(C)**.


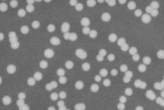

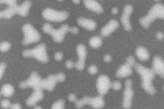

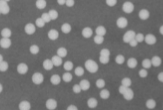

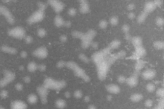

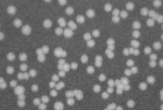

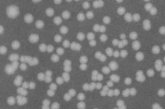

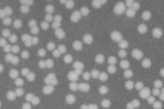

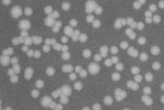


MG1655

ESC19

ESC53

ESC54

37°C

20°C

**Supplementary Figure S5.** Colony morphologies of the strains MG1655, ESC19, ESC53, or ESC54. The overnight cultures of each strain were 10^5^-fold diluted with LB medium. 75 μl of the diluted culture was spread on to LB agar plates. Strains were grown at 37°C or 20°C.
